# Supplementary material for: Discovery and application of insertion-deletion (INDEL) polymorphisms for QTL mapping of early life-history traits in Atlantic salmon
Source: BMC Genomics. 2010 Mar 8;11:156. doi: 10.1186/1471-2164-11-156 (PMC2838853; doi:10.1186/1471-2164-11-156)
Supplement: Additional file 2 — Information on developed 76 locus single-run INDEL panel in Atlantic salmon. Information on fluorescence labeling, primer concentrations, PCR pooling and links to alignments, INDEL motifs and GENESCAN (Burge and Karlin 1997) predictions of genes/exons are available in html format. [file 1471-2164-11-156-S2.ZIP › Additionalfile2/snpsummary3772.html]

```
Cluster 830 Contig 3

prev  Summary    Contig List  next
```

Size of Consensus sequence = 811

Number of sequences = 16

Minimum redundancy = 5

Key

A gi|117860088|gb|EG932784.1|EG932784 EST\_ssal\_evf\_34664 ssalevf mixed\_tissue Salmo salar cDNA Salmo salar cDNA clone ssal\_evf\_546\_048\_rev 5', mRNA sequence  
B gi|24380672|gb|CA050429.1|CA050429 ssalrgb532377 mixed\_tissue Salmo salar cDNA, mRNA sequence  
C gi|117532784|gb|EG864229.1|EG864229 EST\_ssal\_eve\_57098 ssaleve thyroid Salmo salar cDNA Salmo salar cDNA clone ssal\_eve\_577\_310\_rev 5', mRNA sequence  
D gi|117425530|gb|EG757754.1|EG757754 EST\_ssal\_sjb\_6384 ssalsjb mixed\_tissue Salmo salar cDNA Salmo salar cDNA clone ssal\_sjb\_014\_129\_rev 5', mRNA sequence  
E gi|117500607|gb|EG832643.1|EG832643 EST\_ssal\_eve\_44671 ssaleve thyroid Salmo salar cDNA Salmo salar cDNA clone ssal\_eve\_560\_284\_rev 5', mRNA sequence  
F gi|117549699|gb|EG881144.1|EG881144 EST\_ssal\_eve\_24390 ssaleve thyroid Salmo salar cDNA Salmo salar cDNA clone ssal\_eve\_533\_083\_rev 5', mRNA sequence  
G gi|24394713|gb|CA064470.1|CA064470 ssalrgb514197 mixed\_tissue Salmo salar cDNA, mRNA sequence  
H gi|85054041|gb|DW582219.1|DW582219 EST\_ssal\_rgb2\_46638 rgb2 Salmo salar cDNA clone ssal\_rgb2\_575\_152\_fwd 3', mRNA sequence  
I gi|84984815|gb|DW535165.1|DW535165 EST\_ssal\_plnb\_3801 plnb Salmo salar cDNA clone ssal\_plnb\_021\_287\_fwd 3', mRNA sequence  
J gi|24387170|gb|CA056927.1|CA056927 ssalrgb520050 mixed\_tissue Salmo salar cDNA, mRNA sequence  
K gi|89870397|gb|DY726520.1|DY726520 EST\_ssal\_rgb2\_82259 ssalrgb2 mixed\_tissue Salmo salar cDNA Salmo salar cDNA clone ssal\_rgb2\_635\_007\_fwd 3', mRNA sequence  
L gi|85025229|gb|DW553885.1|DW553885 EST\_ssal\_rgb2\_18304 rgb2 Salmo salar cDNA clone ssal\_rgb2\_530\_002\_fwd 3', mRNA sequence  
M gi|24343847|gb|CA042927.1|CA042927 ssalplnb504225 gut Salmo salar cDNA, mRNA sequence  
N gi|85042811|gb|DW570989.1|DW570989 EST\_ssal\_rgb2\_35408 rgb2 Salmo salar cDNA clone ssal\_rgb2\_557\_097\_fwd 3', mRNA sequence  
O gi|117478317|gb|EG810536.1|EG810536 EST\_ssal\_evd\_30802 ssalevd thymus Salmo salar cDNA Salmo salar cDNA clone ssal\_evd\_540\_238\_rev 5', mRNA sequence  
P gi|89845026|gb|DY701149.1|DY701149 EST\_ssal\_rgb2\_56888 ssalrgb2 mixed\_tissue Salmo salar cDNA Salmo salar cDNA clone ssal\_rgb2\_591\_334\_fwd 3', mRNA sequence

2 SNPs detected

A B C D E F G H I J K L M N O P  cosegregation weighted

661 - G - - - - G - - - - - - G G G   2/2 100.00
662 - T - - - - T - - - - - - T T T   2/2 100.00
